# Telomeres in cancer and ageing

**DOI:** 10.1098/rstb.2010.0291

**Published:** 2011-01-12

**Authors:** Luis E. Donate, Maria A. Blasco

**Affiliations:** Telomeres and Telomerase Group, Molecular Oncology Programme, Spanish National Cancer Research Centre (CNIO), Melchor Fernández Almagro, 3, 28029 Madrid, Spain

**Keywords:** telomeres, telomerase, cancer, ageing, shelterins, stem cells

## Abstract

Telomeres protect the chromosome ends from unscheduled DNA repair and degradation. Telomeres are heterochromatic domains composed of repetitive DNA (TTAGGG repeats) bound to an array of specialized proteins. The length of telomere repeats and the integrity of telomere-binding proteins are both important for telomere protection. Furthermore, telomere length and integrity are regulated by a number of epigenetic modifications, thus pointing to higher order control of telomere function. In this regard, we have recently discovered that telomeres are transcribed generating long, non-coding RNAs, which remain associated with the telomeric chromatin and are likely to have important roles in telomere regulation. In the past, we showed that telomere length and the catalytic component of telomerase, Tert, are critical determinants for the mobilization of stem cells. These effects of telomerase and telomere length on stem cell behaviour anticipate the premature ageing and cancer phenotypes of telomerase mutant mice. Recently, we have demonstrated the anti-ageing activity of telomerase by forcing telomerase expression in mice with augmented cancer resistance. Shelterin is the major protein complex bound to mammalian telomeres; however, its potential relevance for cancer and ageing remained unaddressed to date. To this end, we have generated mice conditionally deleted for the shelterin proteins TRF1, TPP1 and Rap1. The study of these mice demonstrates that telomere dysfunction, even if telomeres are of a normal length, is sufficient to produce premature tissue degeneration, acquisition of chromosomal aberrations and initiation of neoplastic lesions. These new mouse models, together with the telomerase-deficient mouse model, are valuable tools for understanding human pathologies produced by telomere dysfunction.

## 1. Role of telomerase in adult stem cells

### (a) Telomeres

Telomeres are ribonucleoprotein complexes at the ends of chromosomes that are essential for chromosome protection and genomic stability. Telomeres consist of tandem repeats of a DNA sequence rich in G bases (TTAGGG in all vertebrates) bound by a six-protein complex known as shelterin. Shelterin encompasses the Pot1-TPP1 heterodimer, the telomere-binding proteins TRF1 and TRF2, and the interacting factors Rap1 and Tin2 [1].

Telomeric chromatin is also enriched in epigenetic marks that are characteristic of constitutive heterochromatin, such as histone tri-methylation and DNA hypermethylation, which act as negative regulators of telomere length and telomere recombination [2].

Telomere shortening below a certain threshold length and/or alterations in the functionality of the telomere-binding proteins can result in loss of telomeric protection, leading to end-to-end chromosome fusions, cell cycle arrest and/or apoptosis. Telomeres also perform other functions, which include the transcriptional silencing of genes located close to the telomeres (this phenomenon is termed subtelomeric silencing), as well as ensuring correct chromosome segregation during mitosis.

### (b) Telomerase

Shortening of telomeres is associated with each round of cell division owing to the inability of conventional DNA polymerases to replicate the ends of linear chromosomes, the so-called ‘end replication problem’. Telomerase is a cellular enzyme capable of compensating this progressive telomere attrition through de novo addition of TTAGGG repeats to the chromosome ends [3]. Telomerase encompasses a catalytic subunit with reverse transcriptase activity (Tert), an RNA component (Terc) that acts as a template for DNA synthesis and the protein dyskerin (Dkc1), which binds and stabilizes Terc.

Robust telomerase expression is a feature of pluripotent stem cells and early stages of embryonic development, although telomerase activity is also present in adult stem cell compartments [4]. Telomerase activity in adult tissues, however, is not sufficient to prevent telomere shortening associated with ageing.

Mutations in the different components of telomerase (Tert, Terc and Dkc1), as well as in some shelterins (Tin2), have been linked to rare human genetic diseases, such as dyskeratosis congenita, aplastic anaemia and idiopathic pulmonary fibrosis [5–8]. These diseases are associated with the presence of short/dysfunctional telomeres and they all exhibit a characteristic failure in the regenerative capacity of tissues (such as the bone marrow) and severe skin hyperpigmentation.

## 2. The telomerase-deficient mouse model

**Figure 1.**
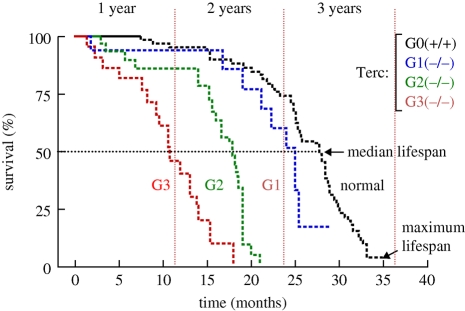
Progressive decrease in median and maximum lifespans along successive generations of telomerase-null mice. Cohorts of successive generations of telomerase-deficient mice (wild-type, black dashed line; first generation G1 Terc^−/−^, blue dashed line; second generation G2 Terc^−/−^, green dashed line; third generation G3 Terc^−/−^, red dashed line) were followed up for a period of 32 months. The figure shows a Kaplan–Meier representation of the survival of the following groups of mice: Terc^+/+^, *n* = 68; G1 Terc^−/−,^ *n* = 17; G2 Terc^−/−^, *n* = 31; G3 Terc^−/−^, *n* = 22. Telomerase-deficient mice have a shorter lifespan than the telomerase-proficient mice, which is further shortened with increasing mouse generations.

Generation of telomerase-deficient mice (Terc knock-out mice) allowed the first demonstration that telomerase is required for telomere maintenance in the context of mammalian organisms, as well as its importance for cancer and ageing. Thus, telomerase-deficient cells exhibit an accelerated telomere shortening that eventually leads to loss of telomere protection and end-to-end chromosome fusions [9–12]. Complete survival curves of increasing generations of telomerase-null mice (G1–G3) [13] indicated that telomere shortening along successive mouse generations [12,14–15] was paralleled by a progressive decrease in both median and maximum longevity (figure 1). Telomerase and telomere maintenance are considered, therefore, to be rate limiting for mouse longevity.

Additionally, telomerase-deficient mice developed premature ageing pathologies with an onset that is anticipated with increasing mouse generations (similarly to the human diseases linked to telomere dysfunction), in agreement with inheritance of shorter telomeres after each generation. Finally, telomerase-deficient mice show increased resistance to cancer, validating telomerase as a promising target for anti-tumour therapies [16].

### (a) Telomerase in adult stem cells

As telomerase expression is restricted to stem cell compartments in the context of the adult organism, it is of interest to determine the impact of telomere shortening on stem cell ageing. Stem cell functionality, measured as the ability of epidermal stem cells to mobilize and regenerate the skin and the hair, is dramatically impaired in telomerase-deficient mice with critically short telomeres (as is the case of G3 telomerase-null mice), a defect that is rescued by either telomerase re-introduction and elongation of short telomeres, or by p53 suppression [17,18]. In this regard, all stem cell defects associated with the presence of critically short telomeres, such as the length of the hair bulb and the thickness of the interfollicular epidermis, are fully rescued in mice doubly deficient for telomerase and p53 when compared with single telomerase-deficient mice. These results identify p53 as an important checkpoint for stem cell and tissue fitness, in a manner that only those stem cells with sufficiently long telomeres will be allowed to regenerate tissues [19].

### (b) Role of telomeres in cancer and ageing: a stem cell-based model

**Figure 2.**
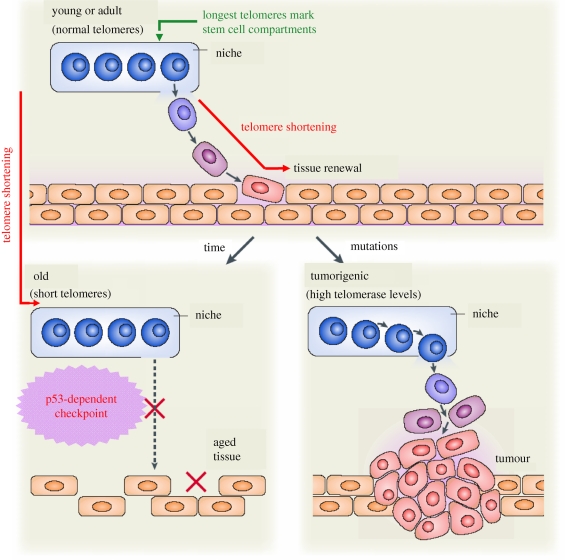
A stem cell-based model for the role of telomeres in cancer and ageing. The longest telomeres mark the stem cell compartments (niches). In young or adult organisms, stem cells (blue rounded cells) repopulate tissues as needed: they exit from the niche, proliferate and differentiate (square orange cells). During this process, stem cells undergo telomere shortening, which is partially counterbalanced by the action of telomerase. In old organisms, stem cell telomeres are too short. Critically short telomeres are recognized as DNA damage, activating a p53-mediated DNA damage signalling response that impairs stem cell mobilization and, as a consequence, the tissue regeneration is suboptimal leading ultimately to organ failure. A decreased stem cell mobilization reduces the probability of accumulating abnormal cells in tissues, providing a mechanism for cancer protection. If the stem cells express aberrantly high levels of telomerase (by acquisition of tumorigenic, telomerase-reactivating mutations), stem cell mobilization is more efficient than normal. Under these higher mobilization conditions, tissue fitness would be maintained for a longer time, increasing lifespan and also the probabilities of initiating a tumour.

We have proposed a stem cell-based model for the role of telomeres in cancer and ageing, which is summarized in figure 2. Adult stem cells reside at specific compartments within tissues, the so-called niches, which are enriched in cells with the longest telomeres [20]. In young or adult organisms with sufficient telomere reserve, adult stem cells efficiently repopulate tissues and repair lesions as needed. In old organisms, however, stem cell telomeres may be too short [20], and this could impair the mobilization of stem cells and the ability to repair tissues efficiently. When telomeres have shortened down to a critical length they are recognized as DNA damage, activating a p53-mediated DNA damage signalling response that prevents the mobilization of the stem cells out of their niches. Decreased stem cell mobilization reduces the probability of accumulating abnormal cells in tissues, thus providing a mechanism for cancer protection. However, the ultimate consequence of impaired mobilization of the stem cells will be organ failure owing to tissue degeneration.

By using mouse models over-expressing telomerase, we and others showed that elevated TERT expression increases stem cell mobilization. Under these conditions of higher mobilization, the fitness of the tissues would be maintained for longer times, therefore increasing the lifespan. The probabilities of initiating a tumour, however, are also higher, especially so if telomerase reactivation occurs in a context of mutations in the tumour suppressors [21].

## 3. Longevity extension by telomerase

**Figure 3.**
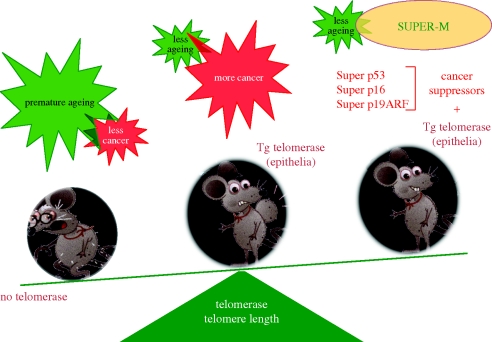
Lifespan extension by telomerase. To address whether telomerase over-expression had an effect on mouse longevity, we first took into account that the absence of telomerase (the Terc knock-out mice) results in premature ageing and also in a lesser incidence of cancer. Second, the constitutive over-expression of telomerase in the epithelia, while attenuating the ageing phenotypes, also increased the incidence of cancer in the epithelia. We generated SUPER-M mice, a mouse model in which the constitutive over-expression of telomerase takes place in a genetic background of increased resistance to cancer achieved by means of over-expression of the tumour suppressors p53, p16 and p19ARF. In the SUPER-M mice, the effects of telomerase on cancer and ageing will be dissociated.

As discussed in §2, the study of telomerase-deficient mice suggests that telomerase is rate limiting for mouse longevity. We next wondered whether telomerase over-expression can extend longevity and, if so, to what extent. We previously observed that while telomerase deficiency results in a lower incidence of cancer [16], constitutive telomerase over-expression results in a slightly increased incidence of cancer (figure 3) [22]. To counterbalance this undesired effect of telomerase over-expression, we over-expressed telomerase in the context of cancer-resistant mice having increased levels of the tumour suppressors p53, p16 and p19ARF (SUPER-M mice; figure 3). In these mice, the effects of telomerase on cancer and ageing will be dissociated, therefore allowing assessment of the role of telomerase on the ageing and fitness of mice.

### (a) SUPER-M mice

Characterization of the SUPER-M mice showed a cancer appearance that was significantly delayed: while in wild-type mice, cancer was detectable at 110 weeks of age (and earlier in those mice over-expressing telomerase), in the SUPER-M mice tumours did not appear until 145 weeks of age [23].

The age for the onset of degenerative lesions is also delayed in SUPER-M mice. In these mice, the symptoms of ageing are also attenuated: for instance, levels of subcutaneous fat are very similar in young and older SUPER-M mice, while the thickness of the subcutaneous fat layer in older wild-type mice is seven times less than that of young wild-type mice. Also, the SUPER-M mice exhibit reduced ageing of the skin; the skin and hair coat of elderly SUPER-M mice are better than of elderly wild-type mice.

Organism ageing of SUPER-M mice is also less: thus neuromuscular fitness is improved in old mice. All the SUPER-M mice successfully passed the neuromuscular coordination test, while not all the wild-type mice were successful in this test, and over half the aged mice failed. SUPER-M mice also had improved glucose tolerance, three times better than that of wild-type mice. SUPER-M mice have longer telomeres than wild-type mice, and this difference in telomere length is much greater in elderly mice. Also, aged SUPER-M mice showed less damage in their telomeric DNA than the aged wild-type mice.

**Figure 4.**
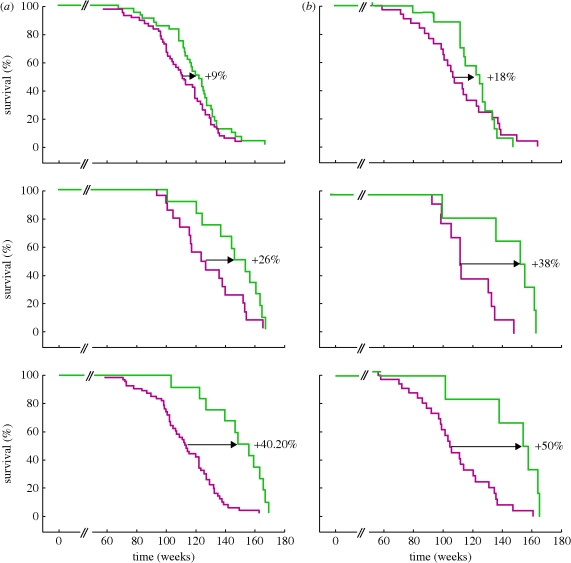
Increased longevity of Sp53/Sp16/SArf/TgTert (SUPER-M) mice. (*a*) Overall survival. Kaplan–Meier representation of the survival curves of the indicated mouse cohorts. Only mice that reached at least up to 50 weeks of age were included. The increase in the median lifespan is indicated. *n* = number of mice per genotype. Statistical significance was assessed using the log-rank test. (*b*) Cancer-free survival. Kaplan–Meier representation of the survival curves of mice of the indicated genotype living for more than 50 weeks excluding those mice that presented malignant tumours at the time of their death (cancer-free survival). The increase in the median lifespan is indicated. *n* = number of mice included in the analysis. Statistical significance was assessed using the log-rank test. (*a*) pink trace, Sp53 (*n* = 68); green trace, Sp53/TgTert (*p* = 0.05; *n* = 56); red trace, Sp53/Sp16/SArf (*n* = 39); green trace, Sp53/Sp16/SArf/TgTert (*p* = 0.05; *n* = 27)(SUPER-M); pink trace, Sp53 (*n* = 68); green trace, Sp53/Sp16/SArf/TgTert (*p* < 0.001; *n* = 27)(SUPER-M). (*b*) pink trace, Sp53 (*n* = 44); green trace, Sp53/TgTert (*p* = 0.47; *n* = 35); pink trace, Sp53/Sp16/SArf (*n* = 33); green trace, Sp53/Sp16/SArf/TgTert (*p* = 0.001; *n* = 22)(SUPER-M); pink trace, Sp53 (*n* = 44); green trace, Sp53/Sp16/SArf/TgTert (*p* = 0.001; *n* = 22) (SUPER-M).

Given the above-described anti-ageing activity of telomerase in the SUPER-M mice, we addressed whether the tranagenic Tert (TgTert) allele had an effect on the lifespan of the cancer-resistant Sp53 and Sp53/Sp16/SArf mice. We obtained survival curves covering the entire lifespan of the Sp53/TgTert and the Sp53/Sp16/SArf/TgTert mice (SUPER-M), compared with Sp53 and Sp53/Sp16/SArf controls; all these mice have the same genetic background composition. We used Sp53 mice as a reference for normal longevity in our mouse cohorts because they have been previously shown to have the same longevity as wild-type mice in two independent studies [24,25]. Analysis of the survival curves indicated a significant extension of median lifespan of 9 per cent and 26 per cent by TgTert expression in the context of cancer-resistant Sp53 and Sp53/Sp16/SArf mice, respectively (figure 4*a*). To further dissociate the effects of TgTert expression on cancer and ageing, we considered separately the lifespan of the cancer-free mice (i.e. those mice that died without malignant tumours; figure 4*b*). The lifespans of this subgroup are determined by ageing and not by cancer, and we observed an even more evident impact of TgTert expression, resulting in a median lifespan extension of 18 per cent and 38 per cent in the Sp53/TgTert and Sp53/Sp16/SArf/TgTert (SUPER-M) mice, respectively, compared with the Sp53 and Sp53/Sp16/SArf controls (figure 4*b*). Furthermore, combined TgTert and Sp53/Sp16/SArf transgenes resulted in a 40.2 per cent extension of the median lifespan when compared with single Sp53 mice (our reference for normal longevity), which was further increased to 50 per cent when considering cancer-free mice (figure 4*b*). We also studied the group of longest lived mice of each genotype to estimate whether TgTert expression had an effect on maximum longevity. The percentage of mice that reached the extremely old age of 3 years was significantly larger for the SUPER-M mice than for their Sp53/Sp16/SArf controls (42% versus 8%), and this extreme survival is further increased when considering cancer-free mice (up to 50%). The mean age of the upper longevity quartile was significantly higher in SUPER-M mice than in their Sp53/Sp16/SArf controls (163 weeks versus 146 weeks). These observations indicate that TgTert expression changes the longevity curve of mice, significantly extending the median lifespan and significantly increasing the percentage of mice that reach extremely old ages. We also wondered whether the observed effects of telomerase on longevity involve its telomere-maintenance activity. To this end, we examined the longevity curve of TgTert mice lacking the RNA component of telomerase (Terc), which is essential for telomerase catalytic activity. We observed that TgTert does not have an effect on the longevity curve of Terc-deficient mice across different generations up to the fourth generation (G2–G4). These results indicate that the anti-ageing activity of Tert requires telomerase catalytic activity, which strongly implicates telomere maintenance as the main mechanism underlying the longevity enhancing effect of telomerase expression.

## 4. Telomere rejuvenation during the generation of induced pluripotent stem cells

To obtain induced pluripotent stem (iPS) cells from adult differentiated cells is a key goal in the development of customized cellular therapies. This type of cell would represent an unlimited source of cells capable of generating all kind of tissues, with the added advantage of avoiding rejection in cell transplantation therapies, as they originate from the same individual. The first approach to nuclear reprogramming was based on nuclear transplantation from somatic cells to enucleated oocytes [26]. Recently, iPS cells have been generated from differentiated cells by the introduction of four transcription factors, Oct4, Sox2, Klf4 and c-Myc [27–28]. However, c-Myc is dispensable in the generation of iPS cells.

**Figure 5.**
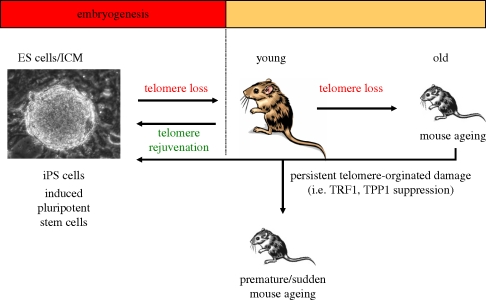
Telomeres and longevity. The mouse embryonic stem cells have very long telomeres which shorten during embryogenesis. Telomere shortening continues during adult life, and is proposed to be a major cause of organism ageing through impairing the regenerative capacity of tissues. Nuclear reprogramming of differentiated cells derived from both young and old mice into induced pluripotent stem (iPS) cells is accompanied by telomerase-dependent telomere elongation. Telomere function is highly dependent on functional shelterin components. Suppression of some shelterins (such as TRF1 and TPP1) results in rapid onset of degenerative pathologies in newborn mice.

As previously stated, telomeres shorten with age, thus contributing to organism ageing by limiting the proliferative capacity of adult stem cells. Although it was known that telomerase activity was augmented in both human and murine iPS cells, it remained unknown whether telomeres re-elongated during nuclear reprogramming and whether the telomeric chromatin would acquire the same characteristics as those of embryonic stem cells (figure 5).

Reprogramming of telomeric chromatin can take place in several possible scenarios: telomerase might not be needed for telomere elongation, telomerase might cooperate with recombination-based mechanisms or, finally, telomere elongation during nuclear reprogramming might completely depend on telomerase activity. We demonstrated that in iPS cells, the telomeres greatly elongate with respect to the telomeres of the parental differentiated cells [29]. This elongation occurs independently of the presence or absence of c-Myc. Furthermore, the efficiency of elongation of telomeres during the generation of iPS cells was the same whether the starting cells were derived from young or elderly individuals. These results, therefore, indicate that telomeres efficiently rejuvenate during nuclear reprogramming of differentiated cells. The elongation of telomeres continues post-reprogramming until they reach the length of the telomeres of the embryonic stem cells. During the reprogramming of telomerase-null cells the telomeres do not elongate. This clearly demonstrates that telomere elongation during nuclear reprogramming of differentiated cells is exclusively mediated by telomerase activity [29].

We also demonstrated that the telomeres of iPS cells acquire the epigenetic marks of the telomeres of embryonic stem cells, among them a low density of trimethylated histones H3K9 and H4K20, and that in the iPS cells there is a loss of telomeric silencing and an increase in the abundance of TERRA (telomeric transcripts) levels [29].

We observed that the reprogramming efficiency of cells derived from increasing generations of telomerase-deficient mice is drastically reduced, and this defect is annulled after telomerase reintroduction [29]. We also observed that the generation of iPS cells needs a minimum telomere length. In fact, when we employed cells derived from the G3 generation of telomerase-deficient mice, reprogramming is impaired, indicating the existence of a minimum required telomeric length for iPS cells to be obtained [29].

## 5. p53 is a key factor limiting reprogramming of suboptimal cells

The fact that cells with short telomeres are not subject to nuclear reprogramming very probably indicates that some ‘reprogramming barriers’ abort the reprogramming of suboptimal parental cells bearing uncapped or dysfunctional telomeres. To explain this observation, we hypothesise low reprogramming rates resulting from the presence of DNA damage in the starting cells. We have demonstrated that the tumour suppressor p53 is a key factor that limits the reprogramming of suboptimal cells, those bearing different kinds of DNA damage such as short telomeres, having deficiencies in their DNA repair systems (ATM- and 53BP1-deficient cells) or exogenously inflicted DNA damage (irradiated cells) [30].

Reprogramming in the face of pre-existing but tolerated DNA damage is aborted by the activation of the p53-dependent DNA damage response (DDR) and apoptosis. p53 suppression allows an efficient reprogramming of cells harbouring persistent DNA damage and chromosomal aberrations. We have observed that during reprogramming, the cells increase their intolerance to the different types of DNA damage and that p53 is critical in avoiding the generation of iPS cells from suboptimal parental cells [30]. Finally, given that certain reprogramming factors promote *in vivo* tumorigenesis, it is tempting to propose that the DDR observed in cultures of p53-deficient cells might be equivalent to the oncogene-induced DDR that takes place during malignant transformation. For the two scenarios nuclear reprogramming and malignant transformation, p53 is critical in controlling the dissemination of damaged cells.

Our results highlight the importance of telomere biology in the generation and functionality of iPS cells, and have important implications for the clinical translation of iPS cell technologies, particularly in patients afflicted with the so-called ‘telopathies’.

## 6. The importance for cancer and ageing of cancelling the dna damage response at chromosome ends

### (a) The shelterin complex

The TTAGGG repeats at the end of mammalian chromosomes associate to a six-protein complex termed shelterin. Shelterin enables cells to distinguish their natural chromosome ends from breaks in the DNA strand by cancelling a DDR at telomeres, as well as regulating telomere length. Telomeres contain additionally a number of proteins that are not part of the shelterin complex, but also have non-telomere-related functions.

The specificity of shelterin for telomeric DNA is through direct recognition of the TTAGGG sequence by three of its components. In particular, TRF1 and TRF2 bind to the double stranded region of telomeric DNA, while Pot1 binds to the TTAGGG repeats of the G-overhang. TRF1 and TRF2 recruit the other four components of shelterin: Tin2 (a TRF1 and TRF2 interacting factor), Rap1, TPP1 and Pot1. These last two proteins form a heterodimer. Shelterin can form a stable complex in the absence of telomeric DNA.

Some mutations in Tin2 as well as genetic variants of TRF1 have been described linked to the rare human diseases dyskeratosis congenita and aplastic anaemia. These conditions [31–33] have as hallmarks epithelial abnormalities, such as skin hyperpigmentation, nail dystrophy and oral leukoplakia.

## 7. Mouse models for conditionally deleted shelterin proteins

### (a) TRF1^*Δ*/*Δ*^ mice

The conventional deletion of the shelterin protein TRF1 in mice is lethal embryonically very early during the blastocyst stage, and for this reason the characterization of the role of TRF1 in differentiated cells has remained impractical.

To study the role of TRF1 in telomere biology and disease in the context of a mammalian organism we generated cells and mice in which TRF1 was conditionally deleted specifically in epithelial (TRF1^*Δ*/*Δ*^ K5-Cre) mice [34]. TRF1 deletion in mouse embryonic fibroblasts (MEFs) did not result in changes in telomere length; on the contrary, it led to a rapid induction of p53/RB-dependent cellular senescence concomitant with the accumulation of abundant damage foci at telomeric DNA. This persistent DNA damage activated phosphorylation of the ATM/ATR kinases and their downstream effectors, the kinases CHK1 and CHK2, resulting in cell cycle arrest. Cells deficient in TRF1 showed abundant end-to-end telomere fusions involving both chromosomes and sister chromatids. We also observed abundant multi-telomeric signals, which are indicative of a high degree of chromosomal fragility that arises from problems in the replication of telomeric DNA [34,35]. Our results demonstrate that TRF1 exerts a protective function against the DDR at telomeres, and that it facilitates the replication of telomeric DNA.

### (b) Consequences of TRF1 deletion in adult stem cells

In accordance with severe telomere dysfunction, TRF1^*Δ*/*Δ*^K5-Cre mice die perinatally and show reduced skin thickness and reduced skin stratification, as well as severe skin hyperpigmentation. Newborn mice also have focal dysplasia in the epithelia of the palate, the non-glandular stomach, oesophagus, tongue and skin. These pathologies are accompanied by activation of a persistent DDR at telomeres, which induces activation of the p53/p21 and p16 pathways resulting in an *in vivo* cell cycle arrest. The latter produces dramatic alterations in the properties of the epithelial stem cells. Thus, morphological development of the hair follicles and the sebaceous glands is completely impaired [34].

### (c) Effect of p53 suppression in TRF1-deficient mice

Suppression of p53 in TRF1^*Δ*/*Δ*^K5-Cre mice rescues perinatal survival (p53^−/−^/TRF1^*Δ*/*Δ*^K5-Cre mice reach four months of age) and the functionality of epidermal stem cells, as these mice grow hair normally and do not exhibit skin hyperpigmentation [34].

Longer lived p53^−/−^/TRF1^*Δ*/*Δ*^K5-Cre mice, however, develop epithelial abnormalities similar to those of human diseases linked to mutations in the components of shelterin and/or telomerase, such as nail atrophy and oral leukoplakia. Furthermore, lack of p53 in p53^−/−^/TRF1^*Δ*/*Δ*^K5-Cre mice leads to the development of spontaneous squamous carcinomas, showing that TRF1 acts as a tumour suppressor by preventing telomere-related genomic instability.

Our results demonstrate that dysfunction of a single telomeric protein is sufficient to produce severe telomeric damage and loss of telomere capping in the absence of telomere shortening, leading to premature tissue ageing, acquisition of chromosomal aberrations and the development of neoplasic lesions.

The *trf1*^*Δ*/*Δ*^ mouse model is of relevance since it constitutes the first mouse model of ageing induced by telomere dysfunction in the absence of critical telomere shortening. This model shows that telomere uncapping and increased telomere fragility do impact on cancer and ageing in the absence of telomere shortening. It also suggests a new class of ‘telopathies’ induced by telomere dysfunction in the presence of normal-length telomeres.

### (d) Tpp1^*Δ*/*Δ*^ mice

The role of TPP1 in telomere regulation *in vivo* and in mouse development and disease has been poorly characterized to date owing to lack of mouse models with complete TPP1 suppression.

We have recently generated *Tpp1*-deficient MEFs as well as mice with targeted *Tpp1* deletion to the stratified epithelia [36]. Both MEFs and mice deleted for *Tpp1* show induction of telomere damage foci and cell cycle arrest, demonstrating that TPP1 protects telomeres from eliciting a DDR. Similarly to TRF1 deficiency, *Tpp*1-null mice die perinatally and show severe skin hyperpigmentation and defective hair follicle morphogenesis. These phenotypes are rescued by p53 suppression, indicating that p53 is a main effector of proliferative defects associated with *Tpp1* deletion.

Unexpectedly, *Tpp1* deletion results in decreased Tert binding to telomeres and accelerated telomere shortening both in MEFs and mice. *Tpp1*-null cells failed to elongate their telomeres when reprogrammed into pluripotent stem cells, a process that is dependent on telomerase activity [29,30], thus indicating that TPP1 is essential for telomere elongation *in vivo*. These results suggest a telomere-capping model where TPP1 not only prevents the induction of a DDR at telomeres by preventing fusions and telomere breakage but also is required for telomere elongation by telomerase.

### (e) Rap1^*Δ*/*Δ*^ mice

Rap1 is a component of the shelterin complex at mammalian telomeres, the *in vivo* role of which in telomere biology has remained largely unknown to date.

We have recently generated cells and mice deleted for Rap1 [37]; mice with *Rap1* deletion in stratified epithelia are viable but had shorter telomeres and developed skin hyperpigmentation at adulthood. We showed that *Rap1* deficiency is dispensable for telomere capping but leads to increased telomere recombination and fragility. We have found that Rap1 binds to both telomeres and to extratelomeric sites. The extratelomeric Rap1-binding sites were enriched at subtelomeric regions, in agreement with preferential deregulation of subtelomeric genes in *Rap1*-deficient cells.

## 8. Telomeres and longevity

The embryonic stem cells of mice have long telomeres, which shorten during embryogenesis (figure 5). Telomere shortening continues throughout the adult life of the mice to such a extent that it has been proposed to be a major cause of mouse ageing since telomere shortening produces defects in the regenerative capacity of tissues.

Nuclear reprogramming of differentiated cells derived from both young and old mice results in the generation of iPS cells. Telomeres rejuvenate in a telomerase-dependent manner during nuclear reprogramming into iPS cells.

Telomere function is highly dependent on functional shelterin components. The suppression of some shelterin proteins (such as TRF1 and TPP1) results in sudden development of degenerative pathologies. Thus, telomere uncapping, even in the presence of normal length telomeres, is capable of inducing age-related pathologies in young mice.

## References

[1] de Lange T. 2005 Shelterin: the protein complex that shapes and safeguards human telomeres. Genes Dev. 19, 2100–2110 10.1101/gad.1346005 (doi:10.1101/gad.1346005) 16166375

[2] Blasco M. A. 2007 The epigenetic regulation of mammalian telomeres. Nat. Rev. Genet. 8, 299–309 10.1038/nrg2047 (doi:10.1038/nrg2047) 17363977

[3] Greider C. W. Blackburn E. H. 1985 Identification of a specific telomere terminal transferase activity in *Tetrahymena* extracts. Cell 43, 405–413 10.1016/0092-8674(85)90170-9 (doi:10.1016/0092-8674(85)90170-9) 3907856

[4] Blasco M. A. 2005 Telomeres and human disease: aging, cancer and beyond. Nat. Rev. Genet. 6, 611–622 10.1038/nrg1656 (doi:10.1038/nrg1656) 16136653

[5] Armanios M. Y. 2007 Telomerase mutations in families with idiopathic pulmonary fibrosis. N. Engl. J. Med. 356, 1317–1326 10.1056/NEJMoa066157 (doi:10.1056/NEJMoa066157) 17392301

[6] Mitchell J. R. Wood E. Collins K. 1999 A telomerase component is defective in the human disease dyskeratosis congenita. Nature 402, 551–555 10.1038/990141 (doi:10.1038/990141) 10591218

[7] Tsakiri K. D. Cronkhite J. T. Kuan P. J. Xing C. Raghu G. Weissler J. C. Rosenblatt R. L. Shay J. W. Garcia C. K. 2007 Adult-onset pulmonary fibrosis caused by mutations in telomerase. Proc. Natl Acad. Sci. USA 104, 7552–7557 10.1073/pnas.0701009104 (doi:10.1073/pnas.0701009104) 17460043 PMC1855917

[8] Vulliamy T. Marrone A. Goldman F. Dearlove A. Bessler M. Mason P. J. Dokal I. 2001 The RNA component of telomerase is mutated in autosomal dominant dyskeratosis congenita. Nature 413, 432–435 10.1038/35096585 (doi:10.1038/35096585) 11574891

[9] Blasco M. A. Funk W. Villaponteau B. Greider C. W. 1995 Functional characterization and developmental regulation of mouse telomerase RNA component. Science 269, 1267–1270 10.1126/science.7544492 (doi:10.1126/science.7544492) 7544492

[10] Blasco M. A. Lee H.-W. Hande P. Samper E. Lansdorp P. DePinho R. A. Greider C. W. 1997 Telomere shortening and tumor formation by mouse cells lacking telomerase RNA. Cell 91, 25–34 10.1016/S0092-8674(01)80006-4 (doi:10.1016/S0092-8674(01)80006-4) 9335332

[11] Lee H.-W. Blasco M. A. Gottlieb G. J. Greider C. W. DePinho R. A. 1998 Essential role of mouse telomerase in highly proliferative organs. Nature 392, 569–574 10.1038/33345 (doi:10.1038/33345) 9560153

[12] Herrera E. Samper E. Blasco M. A. 1999 Telomere shortening in mTR−/− embryos is associated with a failure to close the neural tube. EMBO J. 18, 1172–1181 10.1093/emboj/18.5.1172 (doi:10.1093/emboj/18.5.1172) 10064584 PMC1171208

[13] García-Cao I. García-Cao M. Tomás-Loba A. Martín-Caballero J. Flores J. M. Klatt P. Blasco M. A. Serrano M. 2006 Increased p53 activity does not accelerate telomere-driven ageing. EMBO Rep. 7, 546–552 16582880 10.1038/sj.embor.7400667 PMC1479549

[14] Espejel S. Franco S. Sgura A. Gae D. Bailey S. M. Taccioli G. E. Blasco M. A. 2002 Functional interaction between DNA-PKcs and telomerase in telomere length maintenance. EMBO J. 21, 6275–6287 10.1093/emboj/cdf593 (doi:10.1093/emboj/cdf593) 12426399 PMC137185

[15] Espejel S. Franco S. Rodríguez-Perales S. Bouffler S. D. Cigudosa J. C. Blasco M. A. 2002 Mammalian Ku86 mediates chromosomal fusions and apoptosis caused by critically short telomeres. EMBO J. 21, 2207–2219 10.1093/emboj/21.9.2207 (doi:10.1093/emboj/21.9.2207) 11980718 PMC125978

[16] González-Suárez E. Samper E. Flores J. M. Blasco M. A. 2000 Telomerase-deficient mice with short telomeres are resistant to skin tumorigenesis. Nat. Genet. 26, 114–117 10.1038/79089 (doi:10.1038/79089) 10973262

[17] Flores I. Cayuela M. L. Blasco M. A. 2005 Effects of telomerase and telomere length on epidermal stem cell behavior. Science 309, 1253–1256 10.1126/science.1115025 (doi:10.1126/science.1115025) 16037417

[18] Siegl-Cachedenier I. Flores I. Klatt P. Blasco M. A. 2007 Telomerase reverses epidermal hair follicle stem cell defects and loss of long-term survival associated with critically short telomeres. J. Cell Biol. 179, 277–290 10.1083/jcb.200704141 (doi:10.1083/jcb.200704141) 17954610 PMC2064764

[19] Flores I. Blasco M. A. 2009 A p53-dependent response limits epidermal stem cell functionality and organismal size in mice with short telomeres. PLoS ONE 4, e4934 10.1371/journal.pone.0004934 (doi:10.1371/journal.pone.0004934) 19295915 PMC2654505

[20] Flores I. Canela A. Vera E. Tejera A. Cotsarelis G. Blasco M. A. 2008 The longest telomeres: a general signature of adult stem cell compartments. Genes Dev. 22, 654–667 10.1101/gad.451008 (doi:10.1101/gad.451008) 18283121 PMC2259034

[21] Serrano M. Blasco M. A. 2007 Cancer and ageing: convergent and divergent mechanisms. Nat. Rev. Mol. Cell. Biol. 8, 715–722 10.1038/nrm2242 (doi:10.1038/nrm2242) 17717516

[22] González-Suárez E. Geserick C. Flores J. M. Blasco M. A. 2005 Antagonistic effects of telomerase on cancer and aging in K5-mTert transgenic mice. Oncogene 24, 2256–2270 10.1038/sj.onc.1208413 (doi:10.1038/sj.onc.1208413) 15688016

[23] Tomás-Loba A. 2008 Telomerase reverse transcriptase delays aging in cancer resistant mice. Cell 35, 609–622 10.1016/j.cell.2008.09.034 (doi:10.1016/j.cell.2008.09.034) 19013273

[24] García-Cao I. García-Cao M. Martín-Caballero J. Criado L. M. Klatt P. Flores J. M. Weill J. C. Blasco M. A. Serrano M. 2002 ‘Super p53’ mice exhibit enhanced DNA damage response, are tumor resistant and age normally. EMBO J. 21, 6225–6235 10.1093/emboj/cdf595 (doi:10.1093/emboj/cdf595) 12426394 PMC137187

[25] Matheu A. 2007 Delayed ageing through damage protection by the Arf/p53 pathway. Nature 448, 375–379 10.1038/nature05949 (doi:10.1038/nature05949) 17637672

[26] Campbell K. H. McWhir J. Ritchie W. A. Wilmut I. 1996 Sheep cloned by nuclear transfer from a cultured cell line. Nature 380, 64–66 10.1038/380064a0 (doi:10.1038/380064a0) 8598906

[27] Takahashi K. Yamanaka S. 2006 Induction of pluripotent stem cells from mouse embryonic and adult fibroblast cultures by defined factors. Cell 126, 663–676 10.1016/j.cell.2006.07.024 (doi:10.1016/j.cell.2006.07.024) 16904174

[28] Takahashi K. Tanabe K. Ohnuki M. Narita M. Ichisaka T. Tomoda K. Yamanaka S. 2007 Induction of pluripotent stem cells from adult human fibroblasts by defined factors. Cell 13, 861–872 10.1016/j.cell.2007.11.019 (doi:10.1016/j.cell.2007.11.019) 18035408

[29] Marión R. M. Strati K. Li H. Tejera A. Schoeftner S. Ortega S. Serrano M. Blasco M. A. 2009 Telomeres acquire embryonic stem cell characteristics in induced pluripotent stem cells. Cell Stem Cell 4, 141–154 10.1016/j.stem.2008.12.010 (doi:10.1016/j.stem.2008.12.010) 19200803

[30] Marión R. M. Strati K. Li H. Murga M. Blanco R. Ortega S. Fernandez-Capetillo O. Serrano M. Blasco M. A. 2009 A p53-mediated DNA damage response limits reprogramming to ensure iPS cell genomic integrity. Nature 460, 1149–1153 10.1038/nature08287 (doi:10.1038/nature08287) 19668189 PMC3624089

[31] Savage S. A. Stewart B. J. Weksler B. B. Baerlocher G. M. Lansdorp P. M. Chanock S. J. Alter B. P. 2006 Mutations in the reverse transcriptase component of telomerase (TERT) in patients with bone marrow failure. Blood Cells Mol. Dis. 37, 134–136 10.1016/j.bcmd.2006.07.001 (doi:10.1016/j.bcmd.2006.07.001) 16934504

[32] Savage S. A. Alter B. P. 2008 The role of telomere biology in bone marrow failure and other disorders. Mech. Ageing Dev. 129, 35–47 10.1016/j.mad.2007.11.002 (doi:10.1016/j.mad.2007.11.002) 18160098 PMC2278290

[33] Walne A. J. Vulliamy T. Beswick R. Kirwan M. Dokal I. 2008 TINF2 mutations result in very short telomeres: analysis of a large cohort of patients with dyskeratosis congenita and related bone marrow failure syndromes. Blood 112, 3594–3600 10.1182/blood-2008-05-153445 (doi:10.1182/blood-2008-05-153445) 18669893 PMC2572788

[34] Martínez P. 2009 Increased telomere fragility and fusions resulting from TRF1 deficiency lead to degenerative pathologies and increased cancer in mice. Genes Dev. 23, 2060–2075 10.1101/gad.543509 (doi:10.1101/gad.543509) 19679647 PMC2751970

[35] Sfeir A. Kosiyatrakul S. T. Hockemeyer D. MacRae S. L. Karlseder J. Schildkraut C. L. de Lange T. 2009 Mammalian telomeres resemble fragile sites and require TRF1 for efficient replication. Cell 138, 90–103 10.1016/j.cell.2009.06.021 (doi:10.1016/j.cell.2009.06.021) 19596237 PMC2723738

[36] Tejera A. Stagno d'Alcontres M. Thanasoula M. Marión R. M. Martínez P. Liao C. Flores J. M. Tarsounas M. Blasco M. A. 2010 TPP1 is required for TERT recruitment, telomere elongation during nuclear reprogramming, and normal skin development in mice. Dev. Cell 18, 775–789 10.1016/j.devcel.2010.03.011 (doi:10.1016/j.devcel.2010.03.011) 20493811 PMC3631760

[37] Martínez P. 2010 Mammalian Rap1 controls telomere function and gene expression through binding to telomeric and extratelomeric sites. Nat. Cell Biol. 12, 768–780 10.1038/ncb2081 (doi:10.1038/ncb2081) 20622869 PMC3792482

